# Integrating Assessment of Circadian Rhythmicity to Improve Treatment Outcomes for Circadian Rhythm Sleep-Wake Disorders: Updates on New Treatments

**DOI:** 10.1007/s40675-025-00325-z

**Published:** 2025-02-17

**Authors:** Gabrielle F. Gloston, Katherine C. Ward, G. Carolina Rodriguez-Torres, Karen L. Gamble, S. Justin Thomas

**Affiliations:** 1 Department of Psychology, University of Alabama at Birmingham, Birmingham, AL USA; 2 Department of Psychiatry & Behavioral Neurobiology, Heersink School of Medicine, University of Alabama at Birmingham, 1720 2nd Ave S, Birmingham, AL 35294-0017 USA

**Keywords:** Circadian rhythm sleep-wake disorders, Circadian rhythm assessment, Circadian rhythm treatments, Circadian phase, Entrainment

## Abstract

**Purpose of Review:**

Despite advancements in basic circadian research, development of new diagnostic and treatment strategies for circadian rhythm sleep-wake disorders (CRSWDs) has been slow. Here, we review the most recent innovations in human circadian assessment and emerging new therapies for CRSWDs.

**Recent Findings:**

Researchers have improved existing circadian assessment methods to overcome logistical barriers and developed novel circadian assessment methods. New treatments for CRSWDs involve pharmacological and behavioral treatments that modulate circadian phase, amplitude, and/or robustness of the central circadian clock.

**Summary:**

Commercialization of these emerging tools will require overcoming barriers, such as additional testing to confirm the underlying pathology and mechanism of action of potential treatments. Clinicians and scientists are also called to survey adjacent fields and adopt existing diagnostic tools that may offer diagnostic clarity in CRSWDs. Lastly, we must continue to advocate for medical insurance coverage of current and future tools and technologies to improve patient care.

## Introduction

**Fig. 1:**
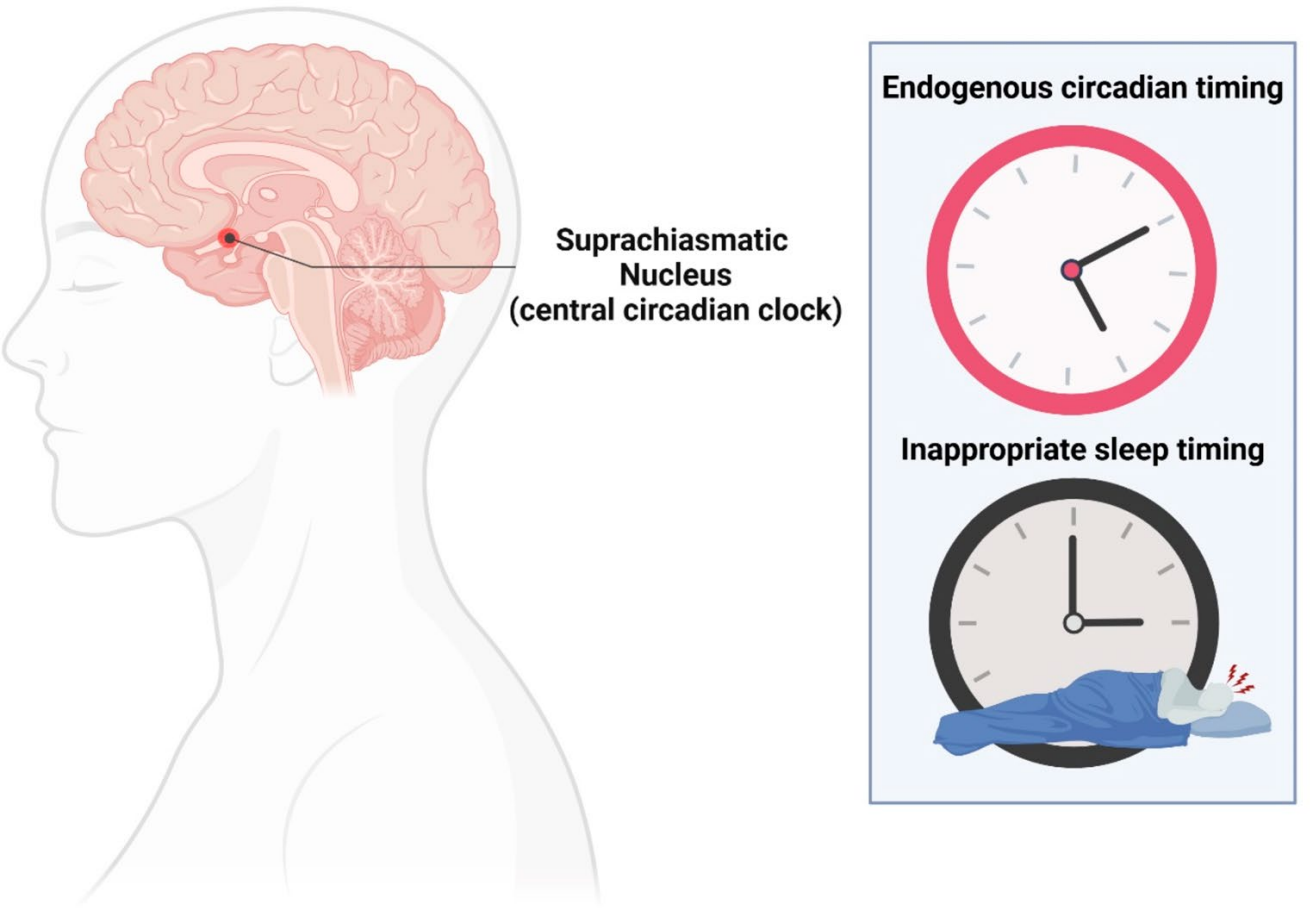
Characterization of intrinsic Circadian Rhythm Sleep-Wake Disorders (CRSWDs). Intrinsic CRSWDs are characterized by inappropriately timed sleep, or an irregular sleep pattern caused by a misalignment between the circadian phase (timing, top right) and the primary sleep period (bottom right). Endogenous circadian timing is driven by the suprachiasmatic nucleus (SCN, left). Symptoms are driven primarily by endogenous circadian pathophysiology but may be exacerbated by behavioral factors. Intrinsic CRSWDs include advanced sleep-wake phase disorder (ASWPD), delayed sleep-wake phase disorder (DSWPD), irregular sleep-wake rhythm disorder (ISWRD), and non-24-hour sleep-wake rhythm disorder (N24SWD). Created in BioRender.com

According to the DSM-5-TR diagnostic criteria, circadian rhythm sleep-wake disorders (CRSWDs) are a class of disorders that involve (1) recurrent sleep disruptions due to either altered circadian rhythms or a misalignment between one’s desired sleep-wake schedule and their internal circadian phase, (2) subsequent excessive sleepiness and/or insomnia, and (3) distress or impairment in daily functioning. This group of disorders can develop due to either endogenous or environmental circadian disruptions, such as malfunctioning of the circadian timing system or chronic shift work, and are categorized as either intrinsic (illustrated in Fig. 1) or extrinsic CRSWDs, respectively [1]. The intrinsic CRSWDs include phase (advanced or delayed), entrainment (non-24 h or irregular), and circadian rhythm disorders not otherwise specified. The extrinsic CRSWDs (i.e., shift work and jet lag disorders) are caused by external influences. This review will focus on intrinsic CRSWDs. The exact prevalence of intrinsic and extrinsic CRSWDs are not known; however, there are an estimated three million people in the U.S. with intrinsic CRSWDs [2]. Delayed Sleep-Wake Phase Disorder (DSWPD) is thought to be the most prevalent CRSWD, particularly in adolescent populations, although the high prevalence may be artificially inflated because it is more disruptive and, therefore, reported at a higher rate than other CRSWDs that are less disruptive to daily life (e.g., Advanced Sleep-Wake Phase Disorder [ASWPD]) [3].

The Sleep Research Society and Society for Research on Biological Rhythms sponsored a joint workshop in 2019 to discuss the current state of intrinsic CRSWDs. The consensus was that despite several advances in basic circadian research, diagnosis and treatment of CRSWDs has not progressed. Specific knowledge gaps about diagnosis and treatment were identified in addition to high-priority research areas for each CRSWD and some common themes emerged. The prevalence, etiology, and clinical course of these disorders remain poorly understood [2]. Further, there is an assumption that the symptoms of CRSWDs can be attributed to a circadian pathophysiological process when there is often no assessment of the circadian clock. Several studies have demonstrated that some individuals diagnosed with phase or entrainment disorders do not exhibit abnormal circadian rhythmicity [4–6]. Up to 40% of individuals diagnosed with DSWPD exhibited what would be a considered a normal (not delayed) circadian phase in melatonin [7]. These studies provide support for the notion that the development of some CRSWDs may have a stronger behavioral or homeostatic component than previously theorized, further highlighting the need for circadian rhythm assessment to disentangle circadian, behavioral, and/or homeostatic contributions to the development of intrinsic CRSWDs. Another major gap in the field that limits the development of precise diagnostic tools and subsequent treatments is the lack of practical biomarkers to evaluate circadian phase and other circadian pathophysiology, such as altered light sensitivity [8]. In this review, we aim to provide a summary of (1) recent developments in human circadian assessment methods and (2) updates on treatments for CRSWDs.

## Updates on Assessment of Circadian Timing and Physiology

Objective assessment of the circadian clock is not required to make a definitive diagnosis based on current diagnostic criteria, and clinical decisions are instead based on a clinical interview combined with actigraphy and/or sleep diaries [9]. Assessment with actigraphy or sleep diaries require 7 to 14 days of monitoring and actigraphy is generally not covered by medical insurance companies [2]. There is a great need for more time-efficient and comprehensive assessments of circadian rhythms to offer more precise and personalized care for those with CRSWDs. Below, we discuss updates on conventional and novel circadian rhythmicity assessment methods.

### Wearables

Actigraphy watches are commonly recommended as an unobtrusive way to objectively measure sleep. The International Classification of Sleep Disorders (ICSD) includes actigraphy measurements in the diagnostic criteria of all CRSWDs as either a recommendation or requirement, with the exception of Jet Lag Disorder [10]. Despite this, there is some contention over whether actigraphy can accurately determine if someone meets the criteria for a CRSWD due to its reliance on sleep timing as measurement [2]. Additionally, some CRSWDs require lengthy sleep monitoring to truly capture the disorder. For example, while ICSD-3 diagnostic criteria for non-24-hour sleep wake disorder (N24SWD) requires only 14 days of actigraphy monitoring, 3 months of sleep diary data are needed to assess changing sleep patterns over time. Capturing true circadian phase is difficult, especially in clinical settings. Dim light melatonin onset (DLMO), as we will discuss in more detail later, is a circadian phase measurement typically gathered with blood or saliva samples spanning six hours prior to and two hours after habitual bedtime in 30- to 60-minute intervals over several hours, which can be challenging to adapt for at-home assessments [11]. Scientists have recently developed a mathematical modeling tool to predict DLMO using actigraphy data, and there is a prototype version publicly available at predictDLMO.com [12, 13]. In a recent study, this tool was used to predict DLMO in shift workers with good concordance to lab-based DLMO (Lin’s concordance coefficient of 0.70) [12]. These results offer a promising outlook for future use of actigraphy as an assessment tool for CRSWDs. While this model has clear clinical implications for extrinsic CRSWDs, this circadian phase estimation tool could also be used to expedite diagnosis or management of intrinsic CRSWDs in a real-world clinical setting. Its successful DLMO prediction in shift workers suggest that it can reasonably predict DLMO in similarly affected clinical populations (e.g., advanced or delayed phase disorders).

Unfortunately, research and clinical-grade wearables may soon lose the scientific community’s favor over consumer wearables with the Philips Actiwatch recently discontinued and insurance providers reluctant to pay for use of actigraphy testing [14]. The American Academy of Sleep Medicine (AASM) is taking steps to advocate for actigraphy in clinical settings with the “Act on Actigraphy,” which urges health insurance companies to provide coverage for actigraphy assessments [15]. Additionally, there may be opportunities for clinicians to advocate for use of actigraphy by including the cost as part of a comprehensive intake and integrative report. Another potential avenue for getting actigraphy assessments in clinic settings is using consumer-grade wearables to which many patients may already have access. Recent studies have examined the feasibility and concordance rates of using consumer-grade wearables such as Fitbit and Apple Watch when compared to research or clinical-grade alternatives with promising results for sleep assessment [16, 17]. However, there are still major concerns about using consumer-grade wearables in clinical or research settings due to the lack of open-access algorithms in how sleep-wake patterns are assessed and therefore a potential lack of standardization across assessments [14]. Consumer wearables also vary in what data they collect, with some only monitoring movement to determine sleep patterns and others adding measurements of light, noise, room temperature, body temperature, oxygen levels, and/or heartrate. Studies comparing consumer wearables to research/clinical grade wearables have been limited and focused primarily on sleep parameters, so the differences in measurement of these variables between devices are largely unknown. For these reasons, it is unclear whether any specific consumer wearable would be a valid option for assessment of circadian rhythms, especially considering predictDLMO.com uses light as its main variable.

### At-home Assessments

DLMO is one of the gold standard methods for estimating human circadian phase; however, gold standard clinical assessments can present many barriers for patients. Similar to wearables, travel costs, long wait times, and financial burden restrict patient access to lab-based DLMO assessments [2]. At-home assessments, which eliminate geographical barriers and reduce the financial burden, may help to improve clinical assessment of human circadian phase [2]. Studies have demonstrated the feasibility and accuracy of at-home DLMO assessments compared to lab-based DLMO assessments in clinical samples [18–21]. For example, DLMO was retrospectively determined in 1,408 (approximately 76%) out of 1,848 participants with suspected DSWPD based on at-home saliva collection [20]. At-home and lab-based DLMO results were significantly correlated in 24 adults with sleep difficulties. Further, the findings demonstrated that the method used to calculate DLMO impacted the strength of this correlation. The absolute threshold method of calculating DLMO in the at-home saliva samples (3 picograms per milliliter, pg/mL) resulted in a stronger correlation with lab-based DLMO results; whereas, the relative threshold method of calculating DLMO (2 standard deviations above the mean) resulted in a weaker correlation, but made it easier to determine DLMO in participants with low melatonin levels [21]. Additional studies have been conducted to further refine the at-home DLMO assessment method by including a measure of light exposure (i.e., wrist actigraphy) and sample timing in the sample collection kits. This addition helped to improve identification of accurate at-home DLMOs in both general and clinical (i.e., DSWPD) participant samples, which again demonstrated high correlation with lab-based DLMOs (*r* = 0.91 and *r* = 0.93, both *p* > 0.001) [18, 19].

A study published in pre-print but not yet peer reviewed demonstrated the feasibility of a self-directed at-home DLMO assessment [22]. This novel study required no in-person visits, and communication with the study team occurred on an as-needed basis. Participants were provided with at-home kits and instructed to complete two separate sample collection periods approximately one week apart. Saliva samples were collected hourly beginning 6 h before the participant’s habitual bedtime and ending 2 h after their habitual bedtime for a total of 9 saliva samples. Data collection also involved wearing the Actiwatch Spectrum Plus for at least 4 weeks [22]. Of the 10 participants, 5 had a CRSWD diagnosis (1 ASWPD, 4 DSWPD) and 5 were controls. DLMO could be calculated twice in 6/10 participants with a correlation of 96% (*p* < 0.0005) between DLMO 1 and 2. DLMO could not be calculated at all in 1 participant diagnosed with ASWPD and 1 participant diagnosed with DSWPD due to consistently low melatonin levels and highly inconsistent sleep times, respectively. DLMO was only calculated for 1 of the 2 sample collections in the remaining 2 participants due to non-compliance. DLMO times calculated from salivary melatonin data were found to be on average 3hrs18min earlier than self-reported sleep start times [22]. Post-DLMO questionnaires were completed by the participants to assess challenges using the at-home assessment. Participants reported difficulties staying awake under the dim light conditions (*n* = 5), staying awake past habitual bedtime (*n* = 1) and remembering to mark an event on the Actiwatch Spectrum Plus (*n* = 3). Other challenges included the length of time required to complete DLMO sample collections and setting up saliva collection space (*n* = 2) [22]. This study suggests that at-home DLMOs are accurate and feasible in assessing underlying physiology associated with CRSWDs. A major limitation of this study is the lack of external validation, such as comparison of at-home DLMO to lab-based DLMO results. While the study does demonstrate that it is possible to assess circadian phase completely remote, the lack of external validation makes it difficult to draw conclusions about accuracy.

A very timely review was recently published summarizing findings on at-home DLMO assessments and more importantly, providing protocol recommendations to support standardization of at-home salivary melatonin sample collection [11]. Recommendations included how to manage external circadian entrainment cues in the home setting, sampling rate, and interpretation of DLMO results. Lastly, the review featured specific guidance on DLMO protocols to assess circadian phase in people with DSWPD [11]. Coordinated efforts between clinicians and scientists are needed to make this a viable clinical assessment tool.

### Lab-based Assessments

Identifying reliable circadian biomarkers and developing practical lab-based assays are critical to advancing the diagnosis and treatment of CRSWDs. Innovative solutions to predict endogenous circadian phase based on multiple blood draws such as TimeSignature bring us closer to this realization of personalized circadian medicine, although there remain some obstacles to overcome before implementing it in a clinical setting [23]. For example, TimeSignature estimates circadian phase within 2 h of DLMO; however, two blood draws taken at least 8 h apart are required for this algorithm to work [23]. An emerging method is TimeMachine, which estimates endogenous circadian phase based on expression of 37 genes from only one blood draw, overcoming the longstanding issue of time constraints in the clinical assessment of circadian rhythmicity [24]. This algorithm also surpasses its predecessor because it has high generalizability across different transcriptional profiling platforms (microarray and RNA sequencing) and does not require multiple iterations of batch correction or retraining. This advantage may increase commercialization given greater applicability to new and old transcriptional profiles [24]. The authors also note that circadian phase prediction does increase with multiple time points (between 8 and 12 h apart); therefore, TimeSignature remains the more accurate prediction algorithm and improves median absolute error by approximately 20–40 min compared to TimeMachine [23]. Still, given the relative accuracy of TimeMachine, Huang and Braun suggest that TimeMachine be used to determine a more precise range of DLMO sampling if endogenous circadian phase is unknown [24]. In this way, using TimeMachine may still be beneficial in a clinical setting to reduce the DLMO sampling window and ultimately reduce time and resources required to assess circadian phase via melatonin onset. Similarly, BodyTime estimates circadian phase based on expression of 12 or fewer genes in human blood monocytes derived from a single blood draw [25]. The assay was externally validated against lab-based DLMO and demonstrated a strong correlation with estimated circadian phases, even among participants with extreme chronotypes. BodyTime’s accuracy and potential clinical utility in assessing and treating CRSWDs is partially attributed to the gene expression platform it uses, NanoString, which has been used for other clinical diagnostic tools that are commercially available [26]. There have also been efforts to estimate circadian phase using whole blood samples. A partial least square regression (PLSR)-based method was tested on 1–2 whole blood samples and resulted in high correlation between predicted and observed circadian phase (*r* = 0.74). Even higher correlations were achieved between predicted and observed circadian phase when two samples were included (*r* = 0.90). The model was initially trained on blood transcriptome data that was collected under various sleeping conditions, including sleeping in or out of phase with an individual’s melatonin and varying degrees of sleep deprivation or sleep debt [27]. This method was limited by the highly controlled clinical research environment, which restricts generalizability [28, 29]. Future studies should recruit more diverse populations, especially those with CRSWDs, which will help scientists evaluate how these transcriptome-based approaches perform in relevant clinical populations.

In addition to transcriptome-based biomarkers and assays, there are also other “omic”-based approaches to estimating circadian phase in humans that could be used in a clinical setting. Metabolomic-based biomarkers and assays have some advantages over other “omic” approaches, including reduced cost, less data processing, and the ability to reflect functional phenotype of individuals [30]. An early metabolomics-based model was developed to predict DLMO based on 58 metabolites and 2 blood samples. This model could estimate circadian phase within a 3-hour range of error. However, this model was limited by a very small, homogenous sample size of young men, making it difficult to generalize to other general and clinical samples [31]. More recent models have been developed using a PLSR approach akin to Laing et al.’s model to estimate both DLMO and dim light melatonin offset (DLMOff) in human plasma samples [27, 32]. The accuracy of the DLMO model was higher than the DLMOff model at baseline; however, there was no statistically significant difference in performance between DLMO and DLMOff prediction across varying conditions (baseline, adequate 9-hour sleep, and 5-hour inadequate sleep). The authors note that while this is the first model to estimate DLMOff, some limitations exist related to the participant sample, highly controlled research environment, and increased food intake during the constant routine protocol. They also highlight a limitation of most metabolomic-based approaches to estimating circadian phase, which is the extent to which metabolites are influenced by food intake and environmental factors. While this approach could provide a quicker alternative to assessing circadian phase in humans compared to traditional lab-based DLMO, there are a number of factors to address that would improve the models’ accuracy, generalizability, and applicability to certain CRSWDs before this type of assay reaches commercialization [32]. Another recently published machine learning model used metabolomic-based data from men and women and highlighted a potential need for sex-based approaches to refine circadian phase estimation. The model used metabolite data derived from 2 to 3 blood samples that were either randomly selected or optimally timed in male and female participants. Interestingly, prediction accuracy significantly improved when more samples were used (up to 3 samples) for male and female participants; however, using more optimally timed samples did not significantly improve prediction accuracy. Food intake served as a major limitation to metabolomic-based models, including Cogswell et al.’s models [30, 32].

Proteomic approaches may be particularly helpful in acknowledging the role of post-translational modifications, which play a key role in regulating the 24-hour cycle of the molecular clock [33–35]. Approximately 1,063 proteins exhibited endogenous circadian rhythmicity in the plasma proteome of 17 healthy adults. Blood samples were collected during a constant routine protocol, which ensures that the group of rhythmic proteins identified were not simply reflective of a 24-hour diurnal pattern [36]. Two independent studies have recently reported changes in the urinary and/or plasma proteome because of sleep deprivation. These studies provide evidence that sleep disruption and perhaps circadian disruption may be associated with a molecular signature in at least two biological sources [37, 38]. Multi-omics profiling may also help to reveal novel molecular interplay and pathways implicated in CRSWDs. For example, a recent study identified new mechanisms for sleep disorders that occur under extreme conditions, such as those in Antarctica. Paired with a characterization of expeditioners’ sleep-wake activity during their year-long station in Antarctica, multi-omic analyses revealed alterations in gene expression, metabolite levels and protein expression that are responsible for various physiological functions [39]. A multi-omic approach to estimating circadian phase may also help to overcome current barriers in the clinical setting.

The development of aptamer-based bioassays is also on the horizon, which allow for detection of biomarkers of the circadian clock, such as melatonin or cortisol in biofluids (i.e., saliva) with high specificity and binding affinity. Aptamers are small, single-strand ribonucleic or deoxynucleic acid molecules that are similar to antibodies but are chemically produced. They are also advantageous in terms of cost and portability, such that they could be used for diagnosis and monitoring of circadian biomarkers associated with CRSWDs [40]. This technique is especially useful for quantification of salivary melatonin, which can exist in very low levels, making it difficult to detect with traditional assays. This would also overcome the cumbersome process of using assays such as radioimmunoassay, which also require specialized training and dedicated space for the proper handling of radioactive isotopes [41–43]. Historically, some barriers to commercialization of aptameric biosensors exist that may be overcome by improving the design of the aptamers [40].

Most techniques that can be used to diagnose CRSWDs primarily involve assessment of circadian phase; however, it is also possible that the circadian dysfunction exists outside of the core circadian clock in the suprachiasmatic nucleus (SCN). Intrinsically photosensitive retinal ganglion cells (ipRGCs), the retinal cells that capture and communicate environmental light level to the SCN, have been shown to mediate the post-illumination pupil response (PIPR), a proxy of these cells’ functioning [44–49]. Abnormal PIPRs have been found in ocular diseases (e.g., glaucoma, macular degeneration, diabetic retinopathy), neurodegenerative diseases (e.g., Parkinson’s Disease, Alzheimer’s Disease), and psychiatric disorders (e.g., Autism Spectrum Disorder, Seasonal Affective Disorder) [50–55]. Researchers have recently demonstrated impairment in PIPR via pupillometry in a small sample of adult participants with DSWPD and sighted N24SWD, which suggests that these patients have difficulty entraining to environmental light cues [8]. Further, melatonin suppression tests may also be used to assess functioning of inputs to the central circadian clock that explain why someone might have altered endogenous circadian phase or difficulty entraining to environmental light cues. It has been demonstrated that melatonin suppression varies greatly from person to person and may offer some value in clinical settings, especially in guiding melatonin supplementation or light therapy specifications [56, 57]. Although this is likely to be used primarily in research settings, a mathematical model has recently been published and made available that supports power analyses for melatonin suppression tests while accounting for variation in light sensitivity and light settings [58]. This innovation is positioned to optimize research, possibly intervention studies for CRSWD patients.

In addition to the central circadian pacemaker in the SCN, peripheral circadian clocks exist throughout the body and coordinate tissue-specific physiological processes important for maintaining health [59–61]. Scientists have demonstrated that internal desynchrony between the central and peripheral clocks can occur due to differences in speed of re-entrainment following circadian disruptions [59, 62–65]. Subsequently, it is unclear if biomarkers that provide estimates of circadian phase are providing information about SCN-driven rhythms or secondary oscillators throughout the body. This is especially important in clinical populations that are more likely to experience circadian misalignment. Advancement in this area of human circadian assessment could lead to more precise estimations of circadian phase and better inform CRSWD treatments.

## Updates on CRSWD Treatments

Due to the challenges discussed above and highlighted in detail in the 2021 workshop report, treatments for CRSWDs are limited [2]. The most recent AASM treatment guidelines for intrinsic CSWDs recommend either bright light therapy, strategically timed administration of exogenous melatonin, or chronotherapy; however, the evidence supporting these treatments is lacking. More specifically, light therapy is recommended to treat ASWPD, and oral administration of exogenous melatonin (in combination with behavioral interventions for children) is recommended to treat DSWPD, although the empirical evidence to support these approaches remain weak [9]. More recently, a study has been published demonstrating positive clinical outcomes in individuals diagnosed with DSWPD after brief light therapy. Compared to the control group, participants who wore the blue light (470 nanometers) glasses for 1–2 h in the morning (between 06:30 − 09:00) for one week experienced significant improvements in sleep quality, nighttime awakenings, and phase shift advance (as indexed by the Morningness-Eveningness Questionnaire) [66]. Light therapy is also recommended by the AASM for Irregular Sleep Wake Rhythm Disorder (ISWRD) in elderly with dementia, and oral administration of melatonin is recommended to treat blind individuals with N24SWD and again, there is only weak empirical evidence in support of these approaches. There are less clear treatment guidelines for sighted individuals with N24SWD due to lack of knowledge of the underlying pathophysiology and paucity of randomized control trials [9]. No new studies have been published investigating possible treatments in sighted individudals with N24SWD to date. The use of exogenous melatonin to treat CRSWDs is complicated by the fact that two studies, one conducted in Canada and the other in the US, have demonstrated that the actual content of over-the-counter melatonin supplements is not accurate, which is concerning given recent reports of increasing reports of melatonin overdose, particularly among children [67, 68].

A recent study demonstrated efficacy of the melatonin receptor agonist (agomelatine) in treating DSWPD [69]. Adolescents and young adults (13–24 years of age) diagnosed with DSWPD were randomly assigned to receive agomelatine with or without cognitive behavior therapy for insomnia (CBT-i) delivered virtually and were assessed via self-report measures before, immediately after completion of treatment, and 8 weeks post-treatment. Regardless of the addition of CBT-i, agomelatine treatment significantly improved sleep as measured by various parameters (sleep onset, sleep offset, sleep duration, insomnia severity index score). There were not many significant differences between groups, suggesting that the most potent component of the intervention was the melatonin receptor agonist [69]. While these results suggest that agomelatine may be of therapeutic benefit for younger individuals with DSWPD, additional studies in larger, more diverse samples are required to provide stronger evidence of effectiveness. Future studies should also include an objective measure of circadian phase (e.g., DLMO, actigraphy) before and after treatment to confirm that symptom relief (i.e., reductions in sleep onset, earlier sleep offset) are in fact due to a significant advance in circadian phase. This may also be beneficial in determining which underlying pathology is most responsive to this treatment.

Aripiprazole, commercially known as Abilify, has been approved as an antipsychotic medication; however, there is evidence it can advance circadian phase in DSWPD. It was hypothesized that this circadian clock-modulating effect was due to the medication’s direct effect on the SCN [70–73]. To test its effects on the central circadian clock, aripiprazole was orally administered to mice after a jet-lag protocol. Mice who were administered aripiprazole exhibited more rapid entrainment to the 6-hr phase advance, and SCN neurons of these mice displayed a dampening of PER2:LUCIFERASE bioluminescence amplitude due to decreased cellular coupling (a weaker circadian network). Taken together, the authors concluded that aripiprazole advances circadian phase via desynchronization of the SCN, allowing the master pacemaker to better adapt to environmental perturbations [74]. Per his commentary, Kitajima points out that although there is evidence suggesting that individuals with certain CRSWDs could potentially benefit from aripiprazole, it is not entirely clear whether its impact stems from direct change in SCN synchronicity. Instead, it remains possible that alternative mechanisms contributed to this change in sleep phase, warranting further in vivo studies [75]. Further, re-entrainment of peripheral clocks in organs throughout the body should be examined given the route of administration of aripiprazole. Together, these studies demonstrate that it is possible that individuals with CRSWDs may find therapeutic benefit from repurposed medications and/or compounds that interact with melatonin receptors in the future in addition to behavioral interventions.

## Conclusions

Diagnosis and treatment of CRSWDs can be challenging due to logistical constraints and knowledge gaps on the underlying pathophysiology; however, there are promising findings in circadian research to improve patient care in this population. Recent literature indicates that there has been a combination of adaptations to existing circadian assessment methods and development of new technologies in service of this mission. Expanding the repertoire of assessment techniques and leveraging relevant research methods can help to accurately identify the etiology and/or contributing factors, leading to more targeted, personalized treatment of CRSWDs (summarized in Table 1). Interdisciplinary science now makes it more possible than ever do this. Some general recommendations to scientists to advance treatment of intrinsic CRSWDs include (1) recruiting patients from more diverse populations to increase generalizability of study results and (2) considering the balance between feasibility, usability and accuracy in the development of novel circadian assessment methods. On the other hand, these diagnostic tools and interventions are of low utility if clinics and patients do not have access to them. Recommendations for clinicians to enhance the treatment of CRSWDs include (1) encouraging patients to participate in relevant research to help improve our understanding of underlying etiologies and (2) advocating for medical insurance coverage of effective diagnostic tools and/or treatments.

**Table 1:** Summary of updates on circadian rhythms assessment and treatments

**Updates in Circadian Rhythm Assessment**				
**Assessment Method**	**Measures**	**Advancement**	**Limitations**	**Ref(s)**
Wrist actigraphy	Physical activity and light	Mathematical model predicts DLMO with high accuracy and generalizability	Light detected by wrist as proxy for light detected by retina remains imperfect	[12, 14]
At-home DLMO	Salivary melatonin	High acceptability and accuracy of at-home method, can include measures of adherence	Potential adherence difficulties and protocol deviations	[14, 18–22]
Transcript-based assays	Gene expression	Circadian phase prediction based on only minimal blood draws (1–2), high generalizability	Lose some accuracy with less time points	[23–25, 27]
Metabolomic-based assays	Metabolite levels	More cost efficient compared to transcript-based assays, requires minimal blood draws (1–3)	Results may be impacted by food intake	[30–32]
Proteomic-based assays	Protein expression, post-translational modifications, or protein-protein interactions	Reflects current physiology, more stable than metabolites	Current assays require samples collected over multiple hours	[36–38]
Aptamer-based bioassays	Quantification of salivary biomarkers (e.g., melatonin)	Can detect biomarkers with higher sensitivity, supports commercialization	Requires further design improvements (e.g., reduce aptamer degradation, make process to select aptamers more efficient)	[40]
PIPR	Intrinsic ipRGC response	Can detect impaired PIPR in DSWPD/N24SWD	Diagnoses of DSWPD based on clinical interview only	[8]
**Updates in Circadian Rhythm Sleep-Wake Disorder Treatment**				
**Treatment**	**Mechanism of Action**	**Advancement**	**Limitations**	**Ref(s)**
Blue light therapy (for DSWPD)	Stimulates ipRGCs, modulates melatonin production	Advance circadian phase and regulate circadian rhythms with portable light therapy	Small sample size, short trial period, long-term efficacy of blue light therapy is unclear	[66]
Agomelatine (for DSWPD)	Melatonin receptor agonist	Advance sleep phase	Small sample size, no DLMO data available	[69]
Aripiprazole/Abilify (for entrainment disorders)	Partial dopamine (D2) and serotonin (5-HT1A) agonist, serotonin (5-HT2A) antagonist	Dampens SCN synchronization, enhances entrainment	Conducted in mice, unclear if results generalize to humans	[74]

DLMO: Dim light melatonin onset, PIPR: pupil illumination, ipRGC: intrinsically photosensitive retinal ganglion cells, PIPR: post-illumination pupil response, DSWPD: delayed sleep-wake phase disorder, N24SWD: non-24 h sleep-wake disorder

## Key References

- Duffy JF, Abbott SM, Burgess HJ, Crowley SJ, Emens JS, Epstein LJ, et al. Workshop report. Circadian rhythm sleep–wake disorders: gaps and opportunities. Sleep. 2021;44(5):zsaa281.
-
  - ◦This workshop report highlights the existing knowledge gaps in intrinsic CRSWDs that need to be addressed to advance clinical practice
- Cheng P, Walch O, Huang Y, Mayer C, Sagong C, Cuamatzi Castelan A, et al. Predicting circadian misalignment with wearable technology: validation of wrist-worn actigraphy and photometry in night shift workers. Sleep. 2021;44(2):zsaa180.
-
  - ◦This study demonstrates an alternative method of circadian phase estimation based on wrist actigraphy in a clinical population
- de Zambotti M, Goldstein C, Cook J, Menghini L, Altini M, Cheng P, et al. State of the science and recommendations for using wearable technology in sleep and circadian research. Sleep. 2023:zsad325.
-
  - ◦This review details recommendations for using wearable technology for tracking features of sleep and the circadian system in light of recent updates in research and consumer wearables
- Bormes G, Love J, Akeju O, Cherry J, Kunorozva L, Qadri S, et al. Self-Directed Home-Based Dim-Light Melatonin Onset Collection: The Circadia Pilot Study. medRxiv. 2023.
-
  - ◦This study demonstrates accuracy of self-directed home-based salivary DLMO sample collection
- Pundir M, Papagerakis S, De Rosa MC, Chronis N, Kurabayashi K, Abdulmawjood S, et al. Emerging biotechnologies for evaluating disruption of stress, sleep, and circadian rhythm mechanism using aptamer-based detection of salivary biomarkers. Biotechnology Advances. 2022;59:107961.
-
  - ◦This study demonstrates the feasibility of circadian phase estimation based on a single blood draw
- Yu Y, Chen Y, Ma L, Qu Y-Y, Li Y-N, Peng Y, et al. Efficacy of agomelatine with cognitive behavioral therapy for delayed sleep-wake phase disorder in young adults: A randomized controlled study. Behavioral Sleep Medicine. 2023;21(5):529-39.
-
  - ◦This study describes the treatment outcomes of portable blue light therapy in DSWPD
- Konishi N, Kumagai H, Sawatari H, Hoshino T, Murase Y, Yamaguchi M, et al. Efficacy of a combination therapy for difficulties waking up in non-school-attending students. Journal of Clinical Medicine. 2022;11(12):3271.
-
  - ◦This study describes an RCT evaluating the effects of combination treatment of a melatonin receptor agonist and cognitive behavioral therapy on DSWPD in young adults
- Kitajima T. Commentary: Aripiprazole disrupts cellular synchrony in the suprachiasmatic nucleus and enhances entrainment to environmental light–dark cycles in mice. Frontiers in Neuroscience. 2024;18:1371195.
-
  - ◦This preclinical study demonstrates potential therapeutic benefits of aripiprazole in the treatment of entrainment disorders

## Data Availability

No datasets were generated or analysed during the current study.
